# Diagnostics of infrapatellar saphenous neuralgia—a reversible cause of chronic anteromedial pain following knee surgery

**DOI:** 10.1007/s00330-021-08184-2

**Published:** 2021-08-03

**Authors:** Schu-Ren Yang, Michael T. Hirschmann, Alain Schiffmann, Balazs K. Kovacs, Julian Gehweiler, Felix Amsler, Anna Hirschmann

**Affiliations:** 1 University Hospital Basel, Clinic of Radiology and Nuclear Medicine, University of Basel, Petersgraben 4, 4031 Basel, Switzerland; 2 Department of Orthopaedic Surgery and Traumatology, Kantonsspital Baselland-Bruderholz, 4101 Binningen, Switzerland; 3 Amsler Consulting Basel, Gundeldingerrain 111, 4059 Basel, Switzerland

**Keywords:** Nerve block, Ultrasound, Saphenous nerve, Knee surgery, Total knee arthroplasty

## Abstract

**Objectives:**

To evaluate the impact of diagnostic nerve block and ultrasound findings on therapeutic choices and predict the outcome after concomitant surgery in patients with suspected neuropathy of the infrapatellar branch of the saphenous nerve (IPBSN).

**Methods:**

Fifty-five patients following knee surgery with suspicion of IPBSN neuralgia were retrospectively included. Ultrasound reports were assessed for neuroma and postsurgical scarring (yes/no). Responders and non-responders were assigned following anesthetic injection of the IPBSN. The type of procedure (neurectomy/interventional pain procedure/other than nerve-associated therapy) and pain score at initial follow-up were recorded and patients were assigned as positive (full pain relief) or negative (partial/no pain relief) to therapeutic nerve treatment. Factors associated with a relevant visual analog scale (VAS) reduction were assessed using uni- and multivariate logistic regression models and chi-square for quantitative and qualitative variables (*p* ≤ 0.05).

**Results:**

Responders (37/55) more often had an entrapment or an evident neuroma of the IPBSN (97% vs. 6%). A positive Hoffmann-Tinel sign (*p* = 0.002) and the absence of knee joint instability (*p* = 0.029) predicted a positive response of the diagnostic nerve block (90%; 26/29). In the follow-up after therapeutic nerve treatment, all patients with full pain relief showed neuromas or entrapment of the IPBSN. Patients negatively responding to therapeutic nerve treatment more frequently showed an additional knee joint instability (25% vs. 4%).

**Conclusion:**

Selective denervation for neuropathic knee pain is beneficial in selected patients with significant VAS reduction after diagnostic nerve block. Non-responders following diagnostic nerve block but sonographic evidence of IPBSN pathologies need to be evaluated for other causes such as knee joint instability.

**Key Points:**

• *Sonographic diagnosis of neuroma or entrapment of the IPBSN is frequently seen in patients with anteromedial knee pain and leads to a good response to diagnostic nerve block following knee surgery.*

• *The vast majority of patients with clinical signs of IPBSN neuropathy and response to a diagnostic nerve block sustained full pain relief following therapeutic nerve treatment.*

• *Patients not responding to therapeutic IPBSN treatment have to be evaluated for other causes of anteromedial knee pain such as knee joint instability.*

## Introduction

**Fig. 1:**
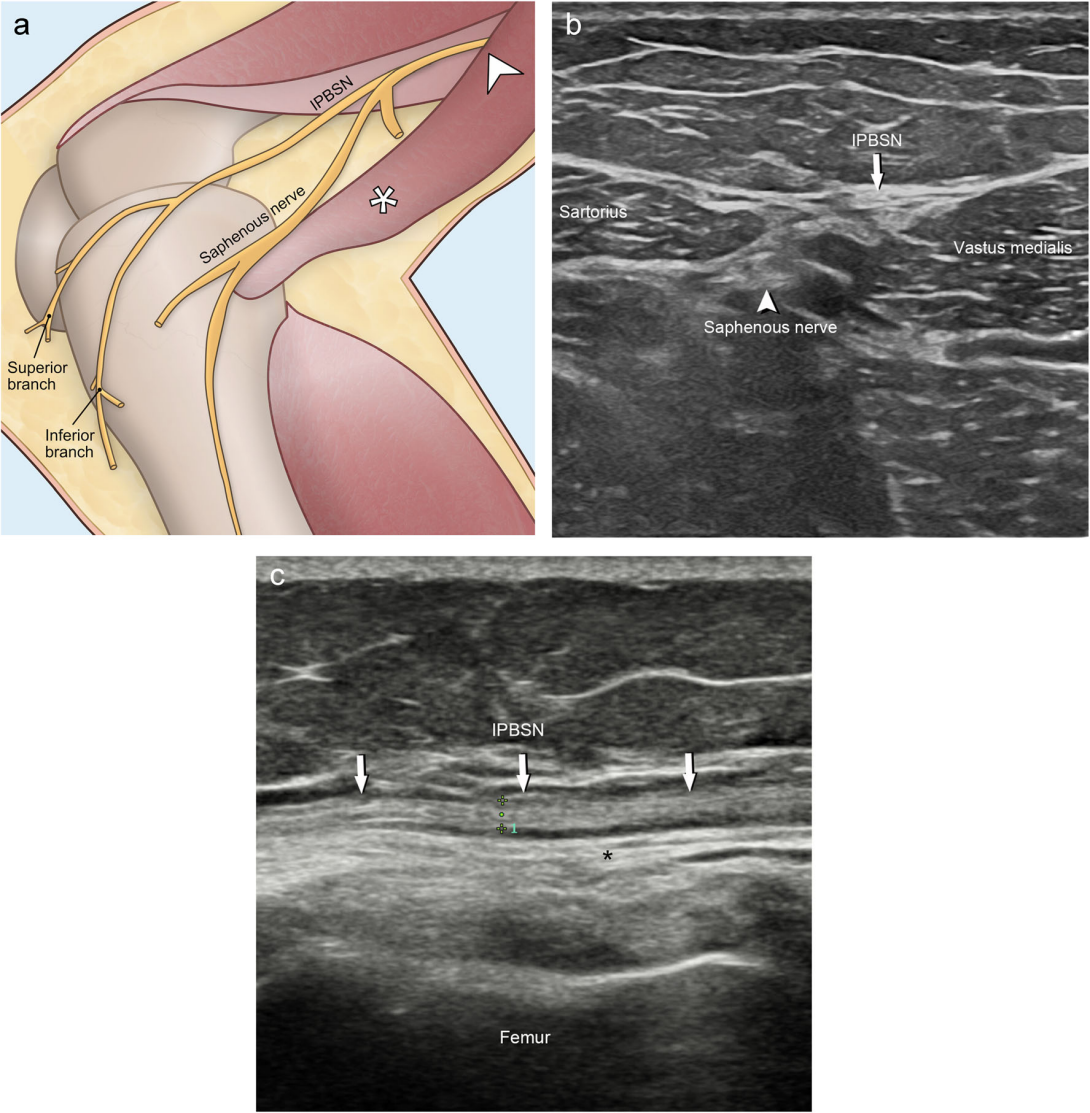
**a** Illustration of the infrapatellar branch of the saphenous nerve (IPBSN) bifurcating into two terminal branches, the superior and inferior IPBSN, innervating the anteromedial skin of the knee, the anterolateral aspect of the proximal lower limb, and the anteroinferior joint capsule. The IPBSN is a pure sensory nerve arising from the saphenous nerve in the subsartorial canal (arrowhead) piercing the fascia anterior or through the sartorius muscle (asterisk). The sartorius muscle (asterisk) is reflected to better demonstrate both the saphenous nerve and the IPBSN. **b** Transverse ultrasound image at the adductor canal shows the IPBSN (arrow) and the saphenous nerve (arrowhead) in between the sartorius and vastus medialis muscles. **c** Longitudinal ultrasound image at the level of the medial femoral condyle shows the IPBSN (arrow) adjacent to the medial collateral ligament (asterisk) in the deep subcutaneous tissue

The infrapatellar branch of the saphenous nerve (IPBSN) is a purely sensory nerve which innervates the anteromedial skin of the knee and the anterolateral aspect of the proximal lower limb, as well as the anterior inferior joint capsule [1, 2]. The IPBSN branches from the saphenous nerve distally to the subsartorial canal, and pierces the deep fascia or sartorius muscle to travel distally in the deep subcutaneous tissue overlaying the medial collateral ligament branching into the superior and inferior IPBSN between the apex of the patella and the tibial tubercle (Fig. 1a).

The IPBSN is target to iatrogenic injuries during knee procedures, such as medial arthroscopic approaches, total knee arthroplasty (TKA), and tendon harvest for anterior cruciate ligament reconstruction. The nerve may be most at risk for damage from the medial retractors that are placed during total knee surgery and a retractor that levers out against the medial tibia, so the nerve may be under tension and a stretch neurapraxia can result.

The incidence of infrapatellar saphenous neuralgia can be found in up to 55–84% of patients after TKA [3–5]. However, clinical symptoms of IPBSN neuropathy may be non-specific and pain following knee surgery, especially after total knee arthroplasties, may be evident in up to 25% of patients with varying etiologies [6–8]. Mochida et al found the incidence of nerve injury, defined by hypoesthesia or anesthesia, to be 22% in patients who had undergone routine arthroscopy [9].

Lesions to this nerve may result in sensorial loss at its innervation territory or painful neuromas at the nerve transection site. Painful neuropathies can also be caused by nerve compression arising from scar adhesions, causing painful entrapment of nerve branches via fibrosis. Physicians have little awareness of its occurrence, resulting in delayed diagnosis as is appropriate therapeutic care. In cases of neuropathic pain, peripheral nociceptors are in a state of continual excitability that induces chemical and anatomical changes in the cortical centers. Factors such as sex, age, genetic susceptibility, and psychosocial context might influence this central process leading to chronic pain [10]. Therapeutic strategies are varied and often involve a multidisciplinary approach. Surgical management of neuropathic pain after peripheral nerve injury with neurolysis and neuroma resection with translocation has been practiced for decades. Percutaneous treatment includes perineural infiltration therapy and pulsed radiofrequency.

As clinical symptoms of IPBSN neuropathy may be non-specific and overlap with other varying causes following knee surgery, we hypothesized that sonographic abnormalities of the IPBSN and response to a diagnostic nerve block correlate with the clinical outcome following nerve treatment.

Therefore, the purpose of this study was to assess the impact of diagnostic IPBSN block results with ultrasound imaging findings on clinical outcomes after concomitant surgery.

## Materials and methods

### Patient selection

The institutional review board of the local ethics committee approved this fully anonymized retrospective study. All patients at least 18 years old with anteromedial knee pain following open and arthroscopic knee surgery, ultrasound and diagnostic nerve block of the IPBSN, and a clinical follow-up between December 2018 and January 2020 were retrieved from the hospital archive from an IPBSN nerve cohort. Included patients were suspected to have neuroma-specific neuropathic pain, including spontaneous pain, electrical spikes, burning pain, allodynia, and hyperalgesia to touch, pressure, or movement. Clinical examination showed a shooting electrical pain when tapping the injured nerve (positive Hoffman-Tinel sign).

In patients with TKA, instability was defined as abnormal and excessive displacement of the articular elements, which has led to clinical failure of the arthroplasty and is known to be one of the most common causes of aseptic failure following TKA [11]. Instability comprised global instability or instability in flexion or extension. Physical examination included observations of gait and stability was assessed with varus-valgus and anterior-posterior stress tests at 30° or 90° flexion, and full extension attempting to reproduce patient symptoms which were complemented using antero-posterior and varus-valgus stress radiographs. Additionally, implant positioning and limb alignment were estimated radiologically.

### Imaging technique and analysis

All diagnostic ultrasound examinations and perineural injections were performed by three radiologists with musculoskeletal subspecialty training. All diagnostic examinations and procedures were performed with a 14-MHz linear-array transducer on LOGIQ S8 XDclear (GE Healthcare). Patients were examined in the supine position with the knee slightly flexed. Ultrasound of the IPBSN and its branches from proximal to distal followed a standardized assessment protocol to identify any structural and perineural abnormality.

First, the transducer was placed transversally at the mid-medial thigh to locate the saphenous nerve in the distal adductor canal. The canal is bordered by the sartorius muscle superficially, the vastus medialis muscle laterally, and the adductor longus muscle deeply [12, 13]. The saphenous nerve runs with the femoral artery and vein inside the canal and exits the adductor canal together with the descending genicular artery. Near the exit of the adductor canal, the saphenous nerve gives off the infrapatellar branch, emerging through the fascial plane interposed between the vastus medialis and sartorius muscle to the subcutaneous layer (Fig. 1b). Then, the IPBSN was traced distally in its subcutaneous, epifascial location (Fig. 1c) until the branch subdivided into one, two, or three terminal branches. In most of our cases, there was a bifurcation with the superior branch running transversely just inferior to the distal pole of the patella and the inferior branch following the medial border of the patellar tendon to the tibial tubercle. If detectable, all branches were followed until their most distally visible part was reached.

**Fig. 2:**
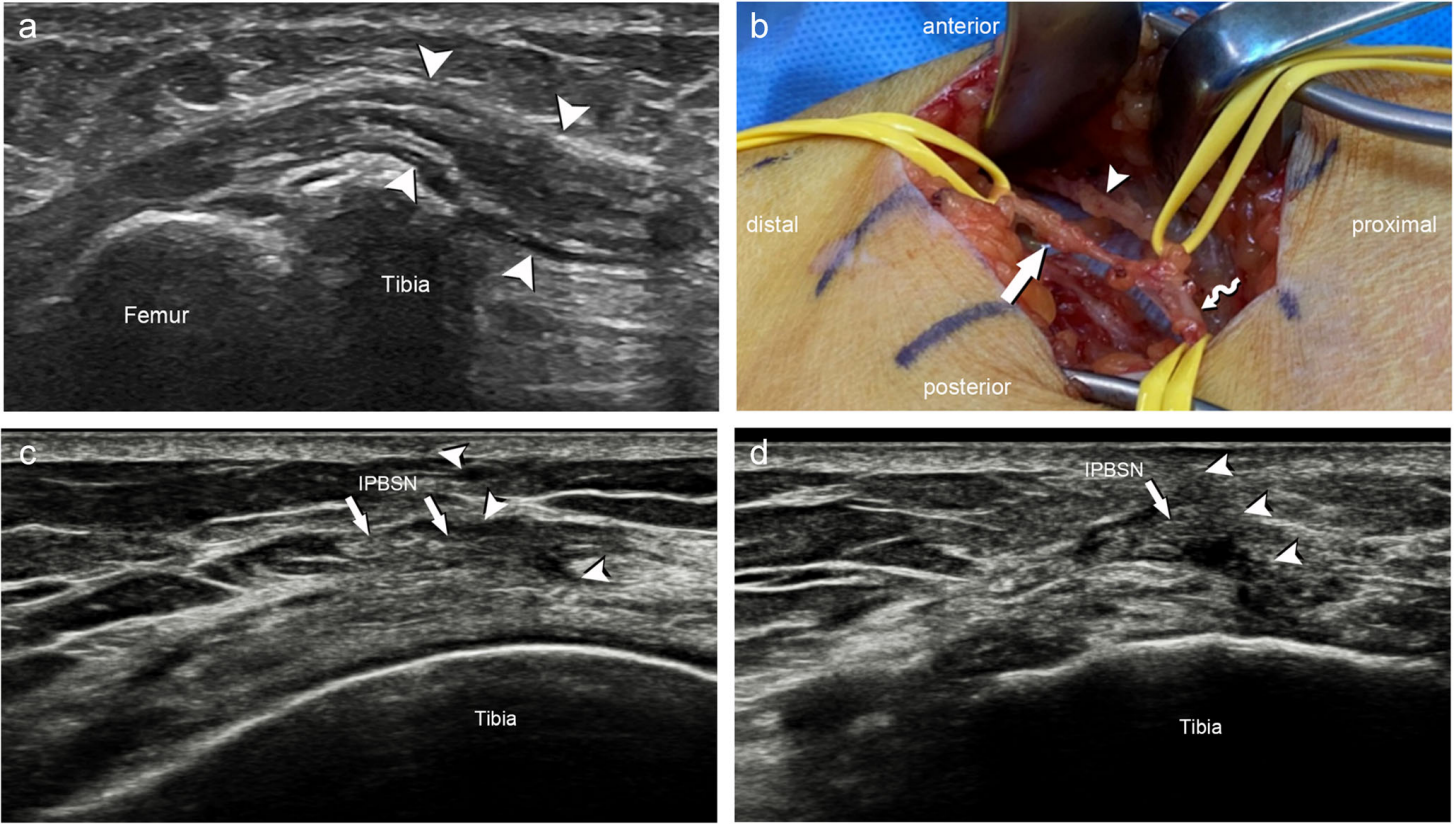
**a** Neuroma-in-continuity of the infrapatellar branch of the saphenous nerve (IPBSN). Longitudinal sonogram shows diffuse hypoechoic enlargement of the inferior branch of the IPBSN (arrowheads) with loss of fascicular architecture. **b** Intraoperative photography of the corresponding neuroma-in-continuity of the inferior branch of the IPBSN (arrow). The IPBSN (wavy arrow) and the superior branch of the IPBSN (arrowhead) are unremarkable. **c**, **d** Entrapment of the inferior branch of the IPBSN (arrows) due to scar tissue (arrowheads) at the level of the tibial tuberosity on transverse (**c**) and longitudinal (**d**) transducer positions

Neuromas were classified as a terminal neuroma or neuroma-in-continuity according to the Seddon and Sunderland classification [14] as a form of ineffective, unregulated nerve regeneration. Basic sonographic findings include focal enlargements with or without the disorganization of the internal fascicular structure indicating a neuroma-in-continuity, or a terminal neuroma in case of nerve transection. One patient with a surgery-proven neuroma-in-continuity of the IPBSN is shown in Fig. 2a and b.

Scar-tethered nerves as defined by Elliot and Sierakowski might cause nerve distortion by shearing, resulting in bursts of pain [15]. In patients with entrapment neuropathy due to overlying scaring, diffuse swelling with decreased echogenicity of the nerve can be sonographically apparent (Fig. 2c, d). The pathophysiological mechanism is thought to be external compression of the nerve causing interference with the intraneural microvasculature, resulting in ischemia or venous congestion, which can lead to epineural edema and increased endoneural fluid [16]. Long-standing scar-tethering with external compression can lead to fibrosis.

Additionally, the nerve size of the IPBSN was measured using diameter (mm) and cross-sectional area (CSA; mm^2^), which was traced within the hyperechoic epineural rim of the nerve with the transducer perpendicular to the nerve.

**Fig. 3:**
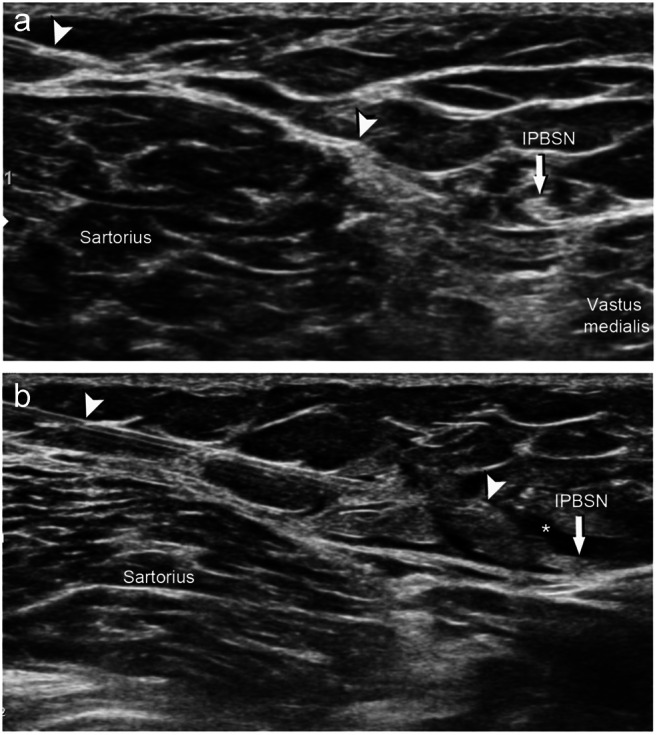
Ultrasound-guided perineural injection of the infrapatellar branch of the saphenous nerve (IPBSN; arrows) using a 25-gauge 60-mm cannula (arrowheads) before (**a**) and after (**b**) injection of 1 mL lidocaine 1% distributing around the IPBSN (asterisk)

When nerve entrapment or a neuroma was identified, the abnormal appearing segment of the nerve was targeted. In cases in which the nerve appeared normal, the injection was directed to the proximal IPBSN (Fig. 3).

Correlation with physical symptoms as tenderness elicited on palpation of the IPBSN (Hoffmann-Tinel sign) or associated paresthesia in the nerve distribution aided in selecting an injection site. Standard sterile techniques were used. Local anesthesia was administered subcutaneously with 1% lidocaine through a 22- or 25-gauge needle, depending on the nerve location depth. In six cases of perineural therapeutic injection, circumferential infiltration around the outer epineurium of the nerve was performed under ultrasound guidance with 1% lidocaine in combination with a water-soluble steroid, dexamethasone (4 mg/mL), in volumes ranging from 1.0 to 5.0 mL.

Pain relief was recorded following a four-step scale using the visual analog scale (VAS; 0–10; 0 = no pain, 10 = severe pain) before and 40 min following diagnostic nerve block: excellent (completely resolved pain according to an absolute VAS reduction to 0 or 1 point or a VAS decrease of > 75%), good (pain decrease of 50–74%), fair (pain decrease of 25–49%), poor (pain decrease of < 25% or increased pain). Patients with excellent and good responses to a diagnostic nerve block were assigned to the responder group, and patients with fair and poor responses were assigned to the non-responder group.

### Clinical report review

A retrospective review of the electronic medical records for patient demographic data, history with details of previous and revision knee surgery, interventional pain procedures (IPP), and follow-up symptomatic relief (full/partial/no pain relief and presence/absence of Hoffmann-Tinel sign) as well as post-procedure complications was performed. Following therapeutic nerve treatment, all patients were assigned into two groups according to their pain relief: positive (full pain relief) and negative (partial and no pain relief).

Precise treatment strategy to surgical or interventional procedures of the IPBSN was determined on a case-by-case basis, depending on the presence of structural abnormalities identified with ultrasound and pain reduction after diagnostic nerve block.

#### Operative management

Surgical intervention on the IPBSN was directed at transection of the nerve proximal to the site of the IPBSN pathology, with implantation of the proximal nerve stump into the adjacent vastus medialis muscle where it was secured with epineural/perineural suture.

#### Interventional pain procedures

Three main IPP, namely peripheral nerve block (PNB), neural therapy, and cryoneurablation, were applied. PNB had been administered as a single injection in this study. Local anesthetics alone in a volume of 1 to 4 mL were used or in conjunction with water-soluble corticosteroids if the goal was longer relief, particularly in the context of neuromas or entrapment neuropathies [17, 18]. Neural therapy is a treatment system to relieve chronic pain and illness through the injection of local anesthetics into scars, peripheral nerves, trigger points, and other tissues. Treatment is based on normalizing the dysfunctional autonomic nervous system, which initiates or propagates many chronic ailments. Cryoneurablation causes Wallerian degeneration due to the loss of the axon through a second-degree freeze. The endoneurium, perineurium, and epineurium remain intact allowing regeneration of the nerve later on [19].

### Statistical analysis

Descriptive data are expressed either as the mean with standard deviation (SD) or n with percentages. Univariate analysis using t-tests for interval-scaled continuous variables and chi-square tests for nominal or ordinal variables was applied to compare responders with non-responders to diagnostic treatment and to compare positively versus negatively reacting patients to therapeutic nerve treatment; *p* ≤ 0.05 was considered statistically significant. All variables with a *p* value ≤ 0.05 were integrated into a multivariate step-by-step forward logistic regression model. Statistical analysis was conducted by using SPSS Statistical Software Package (IBM).

## Results

A total of 59 patients met the inclusion criteria. Of these, four were excluded: two were lost to follow-up and in two patients elective neurectomy had been postponed due to COVID-19. The final cohort included 55 patients (33 women and 22 men) with a mean age of 57.1 ± 13.1 years (range, 18–78 years). The mean time interval between initial surgery and patient symptoms was 6.4 ± 4.9 months. Of those, 34 patients (62%) presented with local allodynia and positive Hoffmann-Tinel sign. Sixty-seven percent (37/55) responded positively to the diagnostic nerve block, while 33% (18/55) did not respond. Responders and non-responders did not differ with respect to demographics or previous surgical procedures. In contrast, non-responders showed a higher rate of joint instability (44 vs. 11%) or intraarticular pathologies including arthrofibrosis or osteoarthritis (56% vs. 19%). Detailed information for both groups is summarized in Table 1.

**Table 1:** Patient demographics and clinical history (total sample, N = 55)

	Responders (n = 37)	Non-responders (n = 18)	*p*
Characteristics			
Age (years)	57.7 ± 13.5	55.8 ± 12.4	0.618
Gender			
Female	21 (57%)	12 (67%)	0.481
Male	16 (43%)	6 (33%)	
Type of prior knee surgery^a^			
Arthroplasty	26 (70%)	16 (89%)	0.127
Arthroscopy	25 (68%)	12 (67%)	0.947
High tibial osteotomy	4 (11%)	3 (17%)	0.541
Additional knee pathology^b^			
Instability	4 (11%)	8 (44%)	0.005
Intraarticular^c^	7 (19%)	10 (56%)	0.006
Extraarticular^d^	10 (27%)	9 (50%)	0.093
Clinical and sonographic examination			
Hoffmann-Tinel sign			
Absent	8 (22%)	13 (72%)	< 0.001
Present	29 (78%)	5 (28%)	
Sonographic IPBSN pathology			
Unremarkable	1 (3%)	17 (94%)	< 0.001
Entrapment	16 (43%)	1 (6%)	
Neuroma	20 (54%)	0 (0%)	
Pain (VAS), mean ± SD			
Pre	6.5 ± 1.9	6.1 ± 1.8	0.422
Post	0.9 ± 1.2	5.0 ± 1.7	< 0.001
Clinical follow-up			
Knee joint surgery and or IPBSN treatment			
Nil	3 (8%)	5 (28%)	< 0.001
Revision arthroplasty	0 (0%)	7 (39%)	
IPP^e^	10 (27%)	6 (33%)	
Neurectomy	20 (54%)	0 (0%)	
Neurectomy and IPP^e^	4 (11%)	0 (0%)	
Time interval			
Time between initial surgery and sonography/diagnostic nerve block (months), mean ± SD	22.0 ± 21.3	29.1 ± 30.5	0.316

Data shown are numerators with percentages in parenthesis, if not stated otherwise

^a^Multiple prior knee surgeries were feasible

^b^Additional knee pathology was obtained on clinical follow-up visits and the final diagnosis; single or multiple diagnoses were possible

^c^Intraarticular pathologies included arthrofibrosis and osteoarthritis

^d^Extraarticular pathologies included iliotibial band and hamstring tendinopathy, muscle imbalance, and pseudoradicular low-back pain

^e^Interventional pain procedure included 14 neural therapies, four peripheral nerve blocks, and two cryoablations

*VAS* visual analog scale, *IPP* interventional pain procedure

### Diagnostic and sonographic findings

Mean caliber of the unremarkable IPBSN was 1.1 ± 0.2 mm (mean CSA, 0.9 ± 0.4 mm^2^), in patients with entrapment 1.4 ± 0.5 mm (mean CSA, 1.8 ± 1.6 mm^2^) and with neuromas was 3.4 ± 1.3 mm (mean CSA, 10.7 ± 8.2 mm^2^).

Responders more often showed Hoffmann-Tinel signs (78 vs. 28%) and more likely had an entrapment or a neuroma of the IPBSN (97% vs. 6%) compared to non-responders. The mean pre-interventional VAS score of all patients was 6.4 (range, 2–10) and post-interventional 2.2 (range, 0–8): while this pain did not differ prior to the examination, it was significantly smaller in the responder group afterwards (VAS 0.9 vs. 5.0).

In a stepwise logistic regression model of the univariately significant variables (except sonographic IPBSN pathology and pain), positive Hoffmann-Tinel signs (*p* = 0.002) and the absence of knee joint instability (*p* = 0.029) were the only criteria to predict positive results of a diagnostic nerve block. The other factors did not reach statistical significance. Table 2 displays the combination of these two factors: 90% of patients with Hoffmann-Tinel sign and knee joint stability, 14% of patients without Hoffmann-Tinel sign and with knee joint instability, and 53% of patients with only one of those two positive criteria responded to the diagnostic nerve block.

**Table 2:** Influence of Hoffman-Tinel sign and knee joint instability on diagnostic nerve block response

Criterion	Responders	Non-responders	Total
No Hoffmann-Tinel sign and joint instability	1 (14%)	6 (86%)	7
Hoffmann-Tinel signs or joint stability	10 (53%)	9 (47%)	19
Hoffmann-Tinel sign and joint stability	26 (90%)	3 (10%)	29
Total	37 (67%)	18 (33%)	55

### Therapeutic treatment

**Fig. 4:**
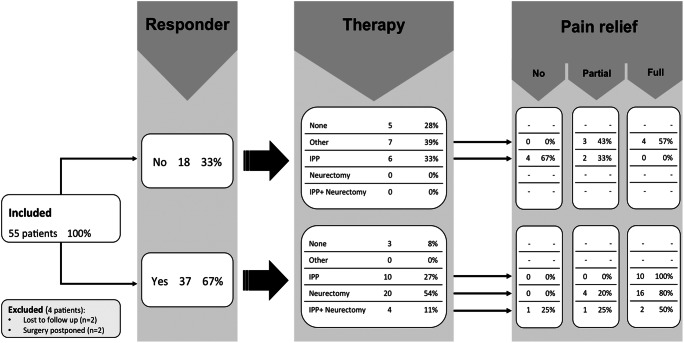
Flowchart demonstrates therapeutic procedures of the infrapatellar branch of the saphenous nerve (IPBSN) and their clinical response following nerve treatment in responding and non-responding patients following diagnostic nerve block. Data are expressed as raw numbers and percentages. Other therapeutics included revision total knee arthroplasty (n = 3), arthrolysis (n = 2), and anterior cruciate ligament reconstruction (n = 1). Mean time interval between IPP and neurectomy in four patients was 2.8 months. IPP interventional pain procedure

A summary of all therapeutic treatment options is illustrated in Fig. 4. Thirty-four responders (92%) received an IPBSN treatment whereas this was the case for six (33%) of the non-responders due to clinical suspicion of possible infrapatellar saphenous neuralgia despite the negative diagnostic nerve block result, comprising a total of 40 patients. Seven non-responders received another therapy (n = 3 revision TKA; n = 2 arthrolysis and n = 1 anterior cruciate ligament reconstruction). Three responders did not undergo nerve treatment: of these, one patient was diagnosed with a TKA instability causing secondary mechanical stress on the IPBSN, which rather required revision surgery to a constrained TKA. Another two responders reported complete pain resolution after diagnostic nerve block at the time of clinical follow-up.

### Response to therapeutic nerve treatment

Of the 40 patients who received a therapeutic nerve treatment (24 women and 16 men with a mean age of 56.6 ± 13.0 years, range 18–78), 28 (70%) sustained full, seven (17.5%) partial, and five (12.5%) no pain relief after treatment (Fig. 4).

For the univariate analysis, patients with negative or partial pain relief were grouped together and compared with those with full pain relief (Table 3). All patients with positive pain relief showed entrapment or neuromas and were responders with lower VAS values following diagnostic nerve block. Additionally, knee joint instability was significantly less frequently evident (4%) in patients with full pain relief as compared to the negative group (25%; *p* = 0.038).

**Table 3:** Patient demographics and clinical history of patients with therapeutic nerve treatment (N = 40)

	Therapeutic nerve treatment response		*p*
	Positive (n = 28)	Negative (n = 12)	
Characteristics			
Age (years), mean ± SD	57.4 ± 14.5	54.8 ± 9.1	0.557
Gender			
Female	16 (57%)	8 (67%)	0.573
Male	12 (43%)	4 (33%)	
Type of prior knee surgery^a^			
Arthroplasty	21 (75%)	9 (75%)	1.000
Arthroscopy	19 (68%)	11 (92%)	0.111
High tibial osteotomy	3 (11%)	1 (8%)	0.818
Additional knee pathology^b^			
Instability	1 (4%)	3 (25%)	0.038
Intraarticular^c^	5 (18%)	4 (33%)	0.283
Extraarticular^d^	6 (21%)	6 (50%)	0.071
Clinical and sonographic examination			
Hoffmann-Tinel sign			
Absent	5 (18%)	3 (25%)	0.605
Present	23 (82%)	9 (75%)	
Sonographic IPBSN pathology			
Unremarkable	0 (0%)	5 (42%)	0.010
Entrapment	13 (46%)	3 (25%)	
Neuroma	15 (54%)	4 (33%)	
Result of diagnostic nerve block			
Non-responder	0 (0%)	6 (50%)	< 0.001
Responder	28 (100%)	6 (50%)	
Pain (VAS), mean ± SD			
Pre	6.5 ± 1.7	6.8 ± 1.8	0.637
Post	0.6 ± 0.9	3.3 ± 1.7	< 0.001
Clinical follow-up			
IPBSN treatment			
IPP^e^	10 (36%)	6 (50%)	0.343
Neurectomy	16 (57%)	4 (33%)	
Neurectomy and IPP^e^	2 (7%)	2 (17%)	
Time intervals			
Time between initial surgery and sonography/ diagnostic nerve block (months), mean ± SD	19.7 ± 17.1	24.0 ± 32.7	0.588
Time between sonography/diagnostic nerve block and neurectomy/IPP^b^ (months), mean ± SD	0.7 ± 0.6	0.8 ± 0.4	0.599
Time between neurectomy/IPP^b^ and clinical follow-up (months), mean ± SD	1.8 ± 1.9	3.4 ± 2.1	0.019

Data are shown are numerators with percentages in parenthesis, if not stated otherwise. Negative responding patients to therapeutic nerve treatment included all patients with partial or no pain relief

^a^Multiple prior knee surgeries were feasible

^b^Additional knee pathology was obtained on clinical follow-up visits and the final diagnosis; single or multiple diagnoses were possible

^c^Intraarticular pathologies included arthrofibrosis and osteoarthritis

^d^Extraarticular pathologies included iliotibial band and hamstring tendinopathy, muscle imbalance, and pseudoradicular low-back pain

^e^Interventional pain procedure included 14 neural therapies, four peripheral nerve blocks, and two cryoablations

*IPBSN* infrapatellar branch of the saphenous nerve, *IPP* interventional pain procedure, *VAS* visual analog scale

**Fig. 5:**
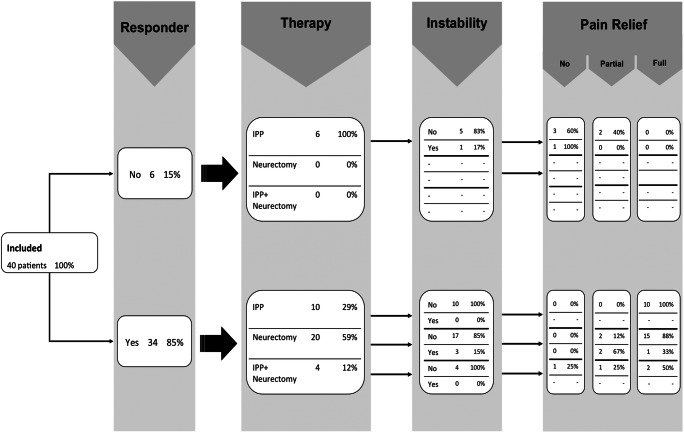
Flowchart demonstrates therapeutic procedures of the infrapatellar branch of the saphenous nerve (IPBSN), additional knee joint instability, and clinical response following nerve treatment in responding and non-responding patients following diagnostic nerve block. Data are expressed as raw numbers and percentages. IPP interventional pain procedure

In a stepwise logistic regression model of the univariately significant variables, the result of sonographic examination (*p* < 0.001) and absence of knee joint instability (*p* = 0.050) were the only ones to predict positive results of nerve treatment. Figure 5 shows the detailed influence of response and knee joint instability on pain relief. Table 4 displays the combination of these two factors: 87% of responders with stable knee joints, 33% of responders with knee joint instability, and none of the non-responders reacted positively on IPBSN treatment.

**Table 4:** Influence of diagnostic nerve block and knee joint instability on infrapatellar branch of the saphenous nerve treatment

Criterion	Therapeutic nerve treatment response		Total
	Positive	Negative	
Non-responder	0 (0%)	6 (100%)	6
Responder and joint instability	1 (33%)	2 (67%)	3
Responder and joint stability	27 (87%)	4 (13%)	31
Total	28 (70%)	12 (30%)	40

Negative responding patients to therapeutic nerve treatment included all patients with partial or no pain relief

## Discussion

Injury to the IPBSN has been reported in the literature, partly due to the frequent use of portals in arthroscopic knee surgery, longitudinal incision for TKA, and ligament harvesting. Indeed, compression or injury of the IPBSN has been recognized and confirmed as a cause of anteromedial knee pain [20] and can result in the formation of a painful neuroma. However, clinical symptoms may be non-specific and difficult to assign to a certain diagnosis. Therefore, we assessed additional etiologies beside neuropathy of the IPBSN and found that non-responders to a diagnostic nerve block showed a higher rate of joint instability or intraarticular pathology including arthrofibrosis or osteoarthritis. On the opposite, responders more often showed Hoffmann-Tinel signs and more likely had an entrapment or a neuroma of the IPBSN.

Our consecutive series of patients evaluated for neuropathy of the IPBSN highlight several critical components of clinical care. First, thorough patient evaluation is mandatory to make the diagnosis as accurate as possible because many of these patients have suffered numerous treatments and surgeries and subjecting them to an additional surgery without sincere expectation of success is probably not in their best interest. The critical step to diagnosis in our study was nerve block using local anesthetic agents as a diagnostic maneuver to confirm clinical suspicion of neuropathic pain rather than interpreting sonographic findings alone. Diagnostic nerve block with an absolute VAS reduction to 1 or 0 points or a VAS decrease of > 50% is feasible to differentiate responders from non-responders (*p* < 0.001). In the vast majority of responders (28/34), neurectomy and/or IPP yielded durable pain relief and functional recovery. Analogous to our diagnostics, the surgical treatment did not vary significantly for compression or axonotmetic injury.

When evaluating patients, it is not essential to distinguish between neuropathic pain due to compression neuropathy caused by scar-tethering or direct injury with neuroma-in-continuity formation bearing in mind the distinct possibility of an associated double crush phenomenon [21, 22].

Despite thorough preoperative evaluation and positive diagnostic nerve block that suggested neuropathy of the IPBSN, one responder did not improve with surgery and continued to have neuropathic pain and functional impairment. Identifying the appropriate nerve involved in pain generation is critical, especially considering overlap of nerve dermatomes and frequent plexus formation between cutaneous nerves [23]. Terminal branches of the IPBSN usually terminate between the patella and the tibial tuberosity by communicating with branches of the medial femoral cutaneous nerve originating from the femoral nerve, thus forming the infrapatellar subsartorial plexus. Deep medial knee pain is predominantly caused by the medial retinacular nerve, a terminal branch of the femoral nerve after it gives off a motor branch to the vastus medialis muscle [24]. One could speculate that this patient who did not respond had pain that was either due to another nerve dermatome or caused by concomitant extraarticular etiologies (medial collateral ligament and muscle deficiency) and the IPBSN was not the predominant contributor.

The five patients that failed surgery with only partial response of up to 50% all had complex medical situations with a history of multiple previous interventions at the knee; however, in many of the patients that successfully responded to surgery, other intra- and extraarticular pathologies and instability were absent. Even though an 82.3% success rate in pain relief is impressive for any therapeutic maneuver in patients with long-standing anteromedial knee pain, the results of this study are consistent with previous published work, stating that 20–30% of neuromas will be refractory to treatment, regardless of type of surgery performed [25, 26].

Six non-responders with clinical suspicion of infrapatellar saphenous neuralgia failed IPP: two patients showed partial and four patients no pain relief, stating that other causes of anteromedial knee pain have to be evaluated.

We recommend using additional selection criteria to stratify responders from non-responders: Hoffmann-Tinel sign in the topography of IPBSN, evident neuroma or scar-tethering on sonography, and evaluation of potential knee joint instability.

The following limitations have to be considered, including a verification bias as patients who were selected to undergo surgery based on the positive diagnostic nerve block result more likely received surgery than those who tested negative. Furthermore, sonography is limited by patient habitus, with degradation of image quality and anatomic detail in obese individuals. Pain evaluation was made using VAS scores alone, which is a subjective outcome measure. The effectiveness of all procedures performed in our unit is evaluated with VAS scores, a relatively reliable, simple, and sufficient way to ascertain the patients` response to a procedure. Finally, although the effect of treatment in the responder group was monitored for more than 2 months, that might not have been a sufficient length of time to determine the long-term effects of the neurectomy or IPP.

Selective denervation for neuropathic postoperative knee pain is beneficial in selected patients with significant VAS reduction after diagnostic nerve block. Non-responders following diagnostic nerve block but sonographic evidence of IPBSN pathologies need to be evaluated for other causes such as knee joint instability.

## Abbreviations

CSA: Cross-sectional area
IPBSN: Infrapatellar branch of the saphenous nerve
IPP: Interventional pain procedure
PNB: Peripheral nerve block
SD: Standard deviation
TKA: Total knee arthroplasty
VAS: Visual analog scale

## Declarations

### Guarantor

The scientific guarantor of this publication is Anna Hirschmann.

### Statistics and biometry

One of the authors has significant statistical expertise.

### Methodology

• Retrospective

• Diagnostic or prognostic study

• Multicenter study
